# The Association Between Body Mass Index and Dental Caries: Cross-Sectional Study

**DOI:** 10.14740/jocmr2433w

**Published:** 2015-12-28

**Authors:** Khaled Alswat, Waleed S. Mohamed, Moustafa A. Wahab, Ahmed A. Aboelil

**Affiliations:** a Department of Internal Medicine, Taif University School of Medicine, Taif, Saudi Arabia; b Dental Clinic, Taif University School of Medicine, Taif, Saudi Arabia

**Keywords:** Body mass index, Caries, DMFT, Habits, Obesity

## Abstract

**Background:**

Obesity is a growing health-related problem worldwide. Both obesity and dental caries are important health issues with multifactorial aspects. Some studies have shown an association between body mass index (BMI) and caries in childhood/adolescence but limited data about such an association are available in adults. The primary goal of this study was to assess the prevalence of dental caries and its relationship to BMI.

**Methods:**

We conducted a cross-sectional study at Taif University Outpatient Clinic, for adults who had a visit to the dental clinic. Baseline characteristics were obtained by the participating physician. The decayed, missing, and filled teeth (DMFT) index was used to determine the prevalence of dental caries. Information about healthy eating, smoking, exercise, sleep patterns, media consumption, and brushing habits were collected.

**Results:**

A total of 385 patients were enrolled with a mean age of 28.39 years, 72.8% were male, mean DMFT index score was 6.55, and 85.5% reported brushing their teeth at least once daily. Of the participants, 55.3% were either overweight or obese, and 42.2% demonstrated a high prevalence of dental caries with no significant difference in BMI when compared to the low dental caries group.

**Conclusions:**

A high prevalence of overweight/obesity and dental caries was observed among the participants. After controlling for potential confounders like smoking and brushing habits, significant positive correlation between BMI and DMFT was observed.

## Introduction

Obesity is defined as a condition of abnormal and excessive fat accumulation in adipose tissue to the extent that health may be adversely affected [1]. It is a global epidemic, and the World Health Organization (WHO) estimates that it is the fifth leading cause of mortality worldwide [2, 3]. Obesity rates have doubled within the last 20 years in many developing and developed countries [4]. Moreover, it is a risk factor for many diseases such as type 2 diabetes, hypertension, hyperlipidemia, cerebrovascular diseases and certain types of cancers [5-8]. The rapid cultural and social changes that have occurred in the Gulf region since the discovery of oil and the subsequent economic boom of the 70s and 80s have been associated with an alarming increase in obesity [9-13]. There are many causes of obesity, but change in the region’s diet quantity and quality is one of the major causes, as it has become more “westernized”. The remarkable economic growth in Saudi Arabia has negatively affected the population’s lifestyle [14]. Although there is a considerable amount of data on obesity prevalence rates, data on dietary habits and food consumption patterns are limited. Recent studies have revealed increasing consumption of animal products and refined foods at the expense of vegetables and fruits [15, 16]. These dietary changes are considered to be one of the potential causes for the observed increase in the prevalence of both overweight and obesity among the Saudi population in the last few decades [17-20].

Dental caries is multi-factorial disease, and affects most of the world population. It is the primary cause of oral pain and tooth loss [21]. It is considered one of the major health-related problems in the young children and one of the most prevalent oral diseases. As a result of its high morbidity potential, this disease is now a main focus of the dental health profession. With its worldwide presence, both genders, all races, all socio-economic status, and all age groups are affected [22]. Because of the discomfort and the financial burden caused by dental caries, prevention is considered an important task for the health profession. Dental disease ranks as the second most expensive disease in Australia (second to cardiovascular disease) and absorbs 6.2% of total recurrent expenditures in health, behind hospital services (39.3%), medical services (18.7%), and medications (14.0%) [23]. Progress is achieved through scientific research that identifies best practices for treating and preventing dental caries.

A decline in dental caries prevalence in developed countries has been observed over the past decades; however, addressing dental caries remains an unmet need for a significant proportion of the world’s population [24, 25]. Recently, caries prevalence has increased in developing nations due to an array of factors, such as intake of sugary foods, low socio-economic status, exposure to fluorides, ethnicity, age, the limited access to oral health services and other lifestyle factors [26]. In 2003, the World Dental Federation, WHO and the International Association for Dental Research issued “Global Goals for Oral Health 2020”. These goals provided guidance for local and national planners and policymakers to improve the oral health status of their populations [27].

Current research in dental medicine trends towards identifying the link between oral health and various systemic diseases. Previous studies have heightened the awareness among dentists about the connection between obesity and oral health in the young [28]. Hence the aim of this study is to assess the prevalence of dental caries, and to identify if there is any relationship between dental caries with age, body mass index (BMI), brushing frequency, dietary habits, physical activity, and smoking in Taif University population.

## Materials and Methods

The study was conducted between June 2014 and May 2015 at Taif University Outpatient Clinics (TUOC), Saudi Arabia. There were no apparent issues that interfered with routine patient care under this protocol, and this study did not directly involve any treatment or intervention. The study included 385 male and female participants who were 18 years or older and who are willing to participate. Data were collected through interview and a self-report questionnaire. The prevalence of dental caries was obtained using the WHO standard criteria for dental caries diagnosis, namely the decayed, missing, and filled teeth (DMFT) index (range 1 - 18), to determine the total number of teeth or surfaces that are decayed, missing, or filled for each study participant. The clinical examination was performed by qualified dentists in sitting chairs in the dental clinic, using natural light and a light source if needed. Caps, masks, gloves, and gauze were used in accordance with infection control guidelines. Weight and height were measured and BMI was calculated according to the formula: weight (kg)/height^2^ (m^2^).

BMI was categorized into underweight (BMI < 18.5), normal (BMI 18.5 - 24.9), overweight (BMI 25 - 30), and obese (BMI > 30). Information about relevant eating habits (i.e., consumption of soft drinks, fast food, and refined sugars), smoking, physical activity, and teeth brushing habits were collected. Brushing frequency was classified as ≥ 2 times daily, once daily, many times weekly but not daily, and weekly or less. We also measured daily media consumption in hours, which included watching TV, using a computer, playing video games, and using a smart phone for entertainment.

### Statistical analysis

Data were analyzed using the Statistical Package for Social Science software, version 20.0 (SPSS, Chicago, IL, USA). The Chi-square test was used to study the relationship between variables and to compare means. Multiple logistic regression analysis was used to determine the degree of association between obesity and dental caries and other variables.

## Results

A total of 385 patients were enrolled in the study. The mean age was 28.39 ± 11.44 years, 72.8% were male, and the mean DMFT index was 6.55. Of the patients, 67.3% reported brushing their teeth ≥ 2 times per day, 18.2% brushed once daily, 7.9% brushed a few times per week, and 6.6% brushed weekly or less; 4.9% of the patients were diabetics, 10.2% were hypertensive, 8.1% were active smokers with mean duration of 1.24 ± 1.24 years, 13.6% reported exposure to passive smoke, 47.1% reported a sedentary lifestyle, 16.9% reported been active ≥ 100 min/week, 33.5% reported soft drinks at least once daily, 45.8% at least eat 1 serving/day of fruit/vegetables, and 30.3% consumed < 3 h of media/day (Table 1).

**Table 1:** Baseline Characteristics of the Whole Cohort

Baseline characteristics	
Mean age (years)	28.39 ± 11.4
Male (%)	72.8
Female (%)	27.2
Hypertension (%)	10.2
Diabetes (%)	4.9
Mean body weight (kg)	75.9 ± 19.99
Mean BMI (kg/m^2^)	26.55 ± 6.3
Mean systolic blood pressure (mm Hg)	122.86 ± 15.2
Mean diastolic blood pressure (mm Hg)	77.23 ± 8.92
Dental health and care	
Mean DMFT index (1 - 18)	6.55
Brushing teeth ≥ 2 times/day (%)	67.3
Brushing teeth once daily (%)	18.2
Brushing teeth few times per week (%)	7.9
Brushing teeth weekly or less (%)	6.6
Special habits	
Active smokers (%)	8.1
Passive smokers (%)	13.6
Sedentary lifestyle (%)	47.1
Active < 100 min/week (%)	36
Active 100 - 300 min/week (%)	11.3
Active > 300 min/week (%)	5.6
Drinking soft drinks at least daily (%)	33.5
Eating 1 serving of fruit/vegetables at least daily (%)	30.3
Media consumptions < 3 h per day	30.3%

The mean body weight of the sample was 75.9 ± 19.99 kg. The mean BMI was 26.55 ± 6.3 kg/m^2^ with 6% of the sample being underweight, 31.4% were overweight, and 23.9% were obese. The mean systolic blood pressure (SBP) was 122.86 mm Hg, and the mean diastolic blood pressure (DBP) was 77.23 mm Hg. We divided the cohort into two groups based on the mean DMFT, so that those with mean DMFT ≥ 6.55 were considered to be in the high dental caries group (HDC) and those with mean DMFT < 6.55 were considered to be in the low dental caries group (LDC). Of the sample, 57.9% were in the LDC group while 42.1% were in the HDC group. Compared to the HDC group, those in the LDC group were non-statically significantly older and had lower body weight/BMI, less likely to be diabetic, but more likely to be hypertensive. Although the LDC group had significantly lower mean DMFT, they also had worse overall brushing habits. The LDC group tended to have lower rates of active smoking, lower rates of daily soft drinks and a comparably sedentary lifestyle rate. HDC groups reported significantly higher rates of eating at least 1 serving of fruit/vegetables per day (Table 2).

**Table 2:** Baseline Characteristics Based on the Dental Caries Group

	LDC group	HDC group	P value
Baseline characteristics			
Mean age (years)	29.3	27.21	0.19
Male (%)	74.89	70.89	0.411
Hypertension (%)	11.9	8.8	0.412
Diabetes (%)	4.3	6.7	0.345
Mean body weight (kg)	75.38	76.9	0.87
Mean BMI (kg/m^2^)	26.2	27.1	0.29
Mean systolic blood pressure (mm Hg)	122.93	122.87	0.14
Mean diastolic blood pressure (mm Hg)	76.54	77.93	0.067
Dental health and care			
Mean DMFT index (1 - 18)	4.08	9.95	< 0.01
Brushing teeth ≥ 2 times/day (%)	66.2	68.9	0.122
Brushing teeth once daily (%)	18.52	18.35	0.366
Brushing teeth few times per week (%)	7.9	8.23	0.35
Brushing teeth weekly or less (%)	7.4	4.4	0.38
Special habits			
Active smokers (%)	7.3	9.4	0.51
Passive smokers (%)	13.8	13.2	0.23
Sedentary lifestyle (%)	46	48.4	0.57
Drinking soft drinks at least daily (%)	30.7	36.7	0.34
Eating 1 serving of fruit/vegetables at least daily (%)	38.9	54.78	0.002
Media consumptions < 3 h per day	29.5	31.84	0.353

Regarding cardiovascular markers, those in the LDC group tended to have non-statically significant lower DBP and comparable SBP. The groups were then divided based on their BMI into normal and overweight/obese. Overweight/obese patients tended to have non-significantly higher DMFT than normal weight patients (mean of 6.65 and 6.47, respectively, P = 0.29), with non-significantly different brushing habits (P = 0.44; Table 3). When comparing the cohort based on gender, the male mean BMI was 26.61 compared to 26.39 in females (P = 0.809), the mean DMFT for males was non-significantly lower (6.46 vs. 6.79, P = 0.38), 9.7% of males were versus 3.8% of females (P = 0.012), and both groups had similar brushing habits. We found a partial positive correlation between the BMI and mean DMFT (P = 0.048), after controlling for diabetes, active smoking, passive smoking, teeth brushing, soft drinks and fruit consumption. However, there was no significant correlation between mean DMFT and media consumption, after controlling for BMI and the other important variables.

**Table 3:** The Mean Decayed/Missing/Filled (DMF) According to BMI Categories

BMI categories	Mean BMI	Mean DMFT
Underweight	17.0	5.26
Normal weight	22.1	6.73
Overweight	27.19	6.42
Obese	34.64	6.48

## Discussion

Given that dental caries rates and BMI both measure diet-related health outcomes; the association between the two is not surprising. Changes to lifestyle and diet since the mid-1990s, for example due to increased affluence and access to high caloric carbohydrate-rich foods and drinks, may help account for the rising prevalence in dental caries and obesity since that time period [29, 30]. Our study showed that 42.1% of participants had high dental caries, in spite of the fact that 85.5% of the study population reported brushing their teeth at least once daily. HDC group were younger and more likely to have higher BMI, drink soda, be an active smoker, and to have sedentary lifestyle. They also had a higher diastolic blood pressure. However, not all studies have found a positive association between BMI and dental caries, as some studies suggest that there is no relationship and others show an inverse relationship [31, 32].

Recent national data from Sweden suggested a positive correlation between dental caries and BMI, and showed that obesogenic behavior such as snacking in early childhood predicted caries development in adolescence [33]. Our study was done in the adult population in Saudi Arabia and evaluated other risk factors for caries like smoking and sugary beverage drinking; even after adjusting for those variables, we found a positive correlation between BMI and dental caries prevalence. To our knowledge, there has been only one systematic review examining the relationship between obesity and dental caries [34]. This review included only seven studies published between 1984 and 2004, five of which only included a pediatric sample. Among them, one found a positive correlation between dental caries and BMI in a sample of 842 children aged 6 - 11 years [35]. Another study found no correlation in a sample of over 5,000 three-year olds [36], while the third was not able to predict future dental caries experience on the basis of BMI status in more than 500 children (aged 5 - 13 years) [37]. Since the publication of this research [34], there has been a lack of studies that assessed the relationship between dental caries and BMI in the adult population in Saudi Arabia and more specifically in Taif city.

Dietary habits are significant contributors to obesity and dental caries epidemics. Changes in dietary pattern like increased consumption of soft drinks, fast food, and refined sugars have led to significant dietary changes among populations, and are considered to be common risk factors for obesity as well as dental caries. Given the strong evidence supporting the association of dental caries with irregular dietary patterns and quality and the fact that abnormal dietary intake has been linked to the development of obesity at a young age, a link between dental caries and body weight is biologically plausible [38]. When children watch a lot of TV, they tend to snack more frequently, particularly on foods that are high in fat and/or sugar. This not only increases their overall caloric intake, which can lead to obesity, but it also increases their risk of developing tooth decay because the amount of time food is in contact with the teeth increases [32].

### Conclusion

More than half of the participants were overweight or obese. Of the participants, 42.2% fell into the high dental caries group. Although the high caries group was generally younger, they had higher DBP and BMI. A significant positive correlation between BMI and DMFT was present, after controlling for the potential confounders like smoking, dietary habits, and brushing habits.

### Practical implications

Dental caries and obesity share some common, modifiable influences such as diet and lifestyle including changes in physical activity and food characters. So obesity can be considered a predictor of dental caries and obese persons need more frequent dental examination and educational care.

### Limitation of the study

Limitations include small sample size and data collection isolated to a single center. Larger studies with a multicenter approach are needed, as are studies with an intervention approach in order to establish causality, and there is need to identify associations and evaluate the weight of each variable as possible risk factors that may have a correlation if we were to have a larger sample size. However, study’s strengths include evaluation of diet, sugary beverage drinking, smoking, and physical activities. We also evaluated some other newly identified potential causes for obesity like media consumption.
